# Membrane Type 1 Matrix Metalloproteinase Regulates Monocyte Migration and Collagen Destruction in Tuberculosis

**DOI:** 10.4049/jimmunol.1403110

**Published:** 2015-06-19

**Authors:** Tarangini Sathyamoorthy, Liku B. Tezera, Naomi F. Walker, Sara Brilha, Luisa Saraiva, Francesco A. Mauri, Robert J. Wilkinson, Jon S. Friedland, Paul T. Elkington

**Affiliations:** * Infectious Diseases and Immunity, Imperial College London, London W12 0NN, United Kingdom;; † National Institute for Health Research Southampton Respiratory Biomedical Research Unit, Clinical and Experimental Sciences Academic Unit, Faculty of Medicine, University of Southampton, Southampton SO17 1BJ, United Kingdom;; ‡ Clinical Infectious Diseases Research Initiative, Institute of Infectious Disease and Molecular Medicine, University of Cape Town, Cape Town 7925, South Africa;; § Department of Histopathology, Imperial College London, London W12 0NN, United Kingdom;; ¶ Department of Medicine, Imperial College London, London W12 0NN, United Kingdom;; ‖ Medical Research Council, National Institute for Medical Research, London NW7 1AA, United Kingdom; and; # Institute for Life Sciences, University of Southampton, Southampton SO17 1BJ, United Kingdom

## Abstract

Tuberculosis (TB) remains a global pandemic and drug resistance is rising. Multicellular granuloma formation is the pathological hallmark of *Mycobacterium tuberculosis* infection. The membrane type 1 matrix metalloproteinase (MT1-MMP or MMP-14) is a collagenase that is key in leukocyte migration and collagen destruction. In patients with TB, induced sputum MT1-MMP mRNA levels were increased 5.1-fold compared with matched controls and correlated positively with extent of lung infiltration on chest radiographs (*r* = 0.483; *p* < 0.05). *M. tuberculosis* infection of primary human monocytes increased MT1-MMP surface expression 31.7-fold and gene expression 24.5-fold. *M. tuberculosis*–infected monocytes degraded collagen matrix in an MT1-MMP–dependent manner, and MT1-MMP neutralization decreased collagen degradation by 73%. In human TB granulomas, MT1-MMP immunoreactivity was observed in macrophages throughout the granuloma. Monocyte–monocyte networks caused a 17.5-fold increase in MT1-MMP surface expression dependent on p38 MAPK and G protein–coupled receptor-dependent signaling. Monocytes migrating toward agarose beads impregnated with conditioned media from *M. tuberculosis*–infected monocytes expressed MT1-MMP. Neutralization of MT1-MMP activity decreased this *M. tuberculosis* network–dependent monocyte migration by 44%. Taken together, we demonstrate that MT1-MMP is central to two key elements of TB pathogenesis, causing collagen degradation and regulating monocyte migration.

## Introduction

New therapeutic approaches are needed to achieve tuberculosis (TB) control, a disease that caused 9.0 million cases and 1.5 million deaths in 2013 (1). Pathology results from the interaction between *Mycobacterium tuberculosis* and the host immune response (2). This pathology is particularly critical in cavitatory pulmonary TB, which results in transmission of infection (3, 4), increased drug resistance (5, 6), morbidity, and mortality (7–9). Improved understanding of host factors that drive lung pathology is required to design novel therapeutic and vaccination strategies (2).

Breakdown of the pulmonary extracellular matrix (ECM) in TB follows inflammatory cellular infiltration and granuloma formation in lung tissue. Secreted matrix metalloproteinases (MMPs) are emerging as key mediators in driving TB pathology. MMPs are zinc-dependent proteolytic enzymes that regulate cellular migration and have the potential to degrade all ECM fibrils (10). MMP-1 causes collagen destruction, whereas the gelatinase MMP-9 plays a critical role in regulating a chemotactic gradient for monocytes toward TB granulomas (11). Membrane-bound MMPs are important in cell migration in diverse inflammatory conditions and can also act as collagenases (12), but their role in TB is poorly understood.

Membrane type 1 MMP (MT1-MMP) was the first membrane-bound MMP to be discovered (13). MT1-MMP degrades collagen in the immediate pericellular environment and is key in cell migration through ECM (14–16). MT1-MMP gene expression has been found to be upregulated in *M. tuberculosis*–infected monocytes (17) and by unbiased analyses of gene expression in TB (18, 19). However, the functional role of MT1-MMP in TB pathogenesis has not been explored. We hypothesized that MT1-MMP may drive inflammatory cell influx to foci of *M. tuberculosis* infection and also contribute to collagen degradation. We demonstrate that MT1-MMP expression is increased in patients with pulmonary TB and is upregulated in primary human monocytes by *M. tuberculosis* infection. MT1-MMP is expressed throughout TB granulomas and is also upregulated by monocyte–monocyte networks. MT1-MMP has dual effects in TB, both causing collagen destruction and also regulating cellular migration, implying a potentially central role in disease pathogenesis.

## Materials and Methods

### Sputum collection and processing

The clinical study was approved by the University of Cape Town Research Ethics Committee (reference no. 516/2011). Written informed consent was obtained in all cases. Participants were prospectively recruited from the Ubuntu clinic in the township of Khayelitsha, Cape Town, South Africa. TB patients met at least one inclusion criteria from sputum smear positive for acid-fast bacillus on microscopy or sputum Gene Xpert positive or sputum culture positive for *M. tuberculosis* or clinical features highly suggestive of TB with diagnostic features on chest radiograph and started on TB treatment by a clinician, conforming to the World Health Organization clinical case definition. Control patients were individuals who were sputum smear and culture negative for *M. tuberculosis* with absent clinical and chest radiograph features of TB. Only HIV negative patients recruited within 72 h of commencing TB treatment were included. Sputum induction, transportation, and mucolysis were performed as described (20). The mucoid layer was filtered by passing through 100-μm pore size strainer (BD Biosciences) and then centrifuged at 500 × *g* for 10 min. The cell pellet was aspirated and 1.5 ml cold TRI reagent (Sigma-Aldrich) added before vortexing. Samples were stored at −80°C before proceeding to RT-PCR analysis. The extent of infiltration on chest radiograph was scored on a scale of 0–10, according to the number of segments involved, using an established scoring system (20).

### Real-time PCR

Sputum was lysed in TRI reagent (Sigma-Aldrich). Total RNA was extracted using phenol/chloroform extraction and the PureLink RNA mini kit (Invitrogen) according to manufacturer’s instructions. RNA was reverse transcribed using the QuantiTect reverse transcription kit (Qiagen) according to the manufacturer’s instructions. RT-PCR was performed on a Stratagene Mx3000Pro machine using the synthesized cDNA, Brilliant II Taq polymerase (Agilent Technologies), and primers/probes (Applied Biosystems) for MT1-MMP and the reference genes β-actin and 18S. The cycle thresholds (Ct) for MT1-MMP and β-actin were used to indicate the relative amount of mRNA in each sample.

### Monocyte purification and stimulation

Monocytes were isolated from single donor leukocyte cones (National Health Service Blood and Transfusion, London, U.K.) or fresh blood from healthy donors by density gradient centrifugation over Ficoll-Paque (GE Healthcare Life Sciences) and adhesion purification. Monocyte purity was >95% by flow cytometry and viability was >95% by trypan blue exclusion assay. Monocytes were plated in tissue culture plates or chamber slides. Experiments were commenced immediately using RPMI 1640 supplemented with 2 mM glutamine, 10 μg/ml ampicillin, with 10% heat-inactivated FCS (Biowest) in a humidified incubator supplemented with 5% CO_2_ at 37°C. Stimuli used were direct infection with *M. tuberculosis* strain H37Rv or conditioned medium from *M. tuberculosis*–infected monocytes (CoMTb).

### *M. tuberculosis* culture

*M. tuberculosis* H37Rv was cultured in Middlebrook 7H9 medium (BD Biosciences, Oxford, U.K.). *M. tuberculosis* in mid-log growth at an OD of 0.60 (Biowave cell density meter, WPA) was used for infecting monocytes immediately after adhesion purification at a multiplicity of infection (MOI) of 1.

### Preparation of CoMTb

Monocytes were plated in RPMI 1640 supplemented with 2 mM glutamine and 10 μg/ml ampicillin and immediately infected with *M. tuberculosis* at an MOI of 1. After 24 h, the cell culture medium was aspirated and filtered through a 0.2-μm Anopore membrane (Whatman) to remove live *M. tuberculosis* and MMPs (21). The resulting cell culture supernatant is termed CoMTb and contains an *M. tuberculosis*–induced intercellular network of cytokines, chemokines, and growth factors. This was used to stimulate monocytes at a 1:5 dilution immediately after adhesion purification. Conditioned medium from control monocytes is the cell culture supernatant from uninfected monocytes after 24 h of incubation.

### Flow cytometry

Adhesion-purified monocytes were harvested from tissue culture plates using cell dissociation buffer (enzyme-free) (Invitrogen) at 37°C for 15 min and a cell scraper. Cells were centrifuged at 1200 rpm for 5 min for pelleting and blocked with 10% heat-inactivated human serum/1% BSA, then stained with a PE-conjugated mouse anti–MT1-MMP Ab (R&D Systems, FAB9181P). When cells had been infected with *M. tuberculosis*, they were then fixed in 2% paraformaldehyde. Flow cytometry was performed immediately after staining on a FACSCalibur (BD Biosciences). Live cells were gated, confirmed by propidium iodide staining. Harvested monocytes were identified for gating on the forward scatter/side scatter plot. Negative controls were unstained, unstimulated monocytes. FlowJo software (version 7.6.2) was used for data analysis.

### Western blotting

Monocytes were harvested into SDS lysis buffer (62.5 mM Tris, 2% SDS, 10% glycerol, 50 mM DDT, 0.01% bromphenol blue; Sigma-Aldrich), denatured at 90°C, loaded on a NuPAGE 4–12% Bis-Tris gel (Invitrogen), and separated by electrophoresis at 200 V for 50 min in MOPS running buffer (Invitrogen). Proteins were electrotransferred to a nitrocellulose membrane (GE Healthcare Life Sciences) at 30 V for 90 min, blocked with 0.1% Tween 20 (Sigma-Aldrich)/5% nonfat milk for 1 h, and then incubated with primary Ab overnight. Detection was by HRP-conjugated secondary Ab and chemiluminescent substrate (ECL Plus Western blotting detection system, GE Healthcare Life Sciences). Densitometry was analyzed using Image J software version 1.44p (National Institutes of Health, Bethesda, MD). For MT1-MMP Western blots, the primary Abs were rabbit anti–MT1-MMP (Millipore, AB 6004) and mouse anti–β-actin (Sigma-Aldrich, A1978). The secondary Abs were HRP-conjugated goat anti-rabbit (Cell Signaling Technology) and goat anti-mouse (Jackson ImmunoResearch Laboratories). For phosphorylated Western blots, the primary Abs were to phosphorylated and total p38/ERK MAPK (Cell Signaling Technology). The secondary Ab was HRP-conjugated goat anti-rabbit (Cell Signaling Technology).

### Immunofluorescent microscopy and fluorescent collagen degradation assay

Monocytes in chamber slides (PAA Laboratories) were blocked with 5% human serum/1% BSA for 1 h and stained with mouse anti MT1-MMP (Millipore, MAB3328), goat anti-mouse Alexa Fluor 647–conjugated secondary Ab (Invitrogen, A-21235), and DAPI nuclear stain (Invitrogen). Permeabalization was performed with 0.5% Triton X-100 (Merck) and fixation with 2% paraformaldehyde. For the fluorescent collagen degradation assay, chamber slides were coated with FITC-conjugated type I collagen from bovine skin (Sigma-Aldrich), prior to adhering the monocytes using a modification of a previously described technique. Poly-l-lysine (0.005%) (Sigma-Aldrich) was first used to create a substratum to improve cell adherence, then 0.5% glutaraldehyde (BDH) to covalently couple the substratum to the fluorescent collagen. This created a thin two-dimensional collagen matrix for detection of pericellular collagen degradation by loss of fluorescent signal. Fluorescent microscopy was performed on a Leica TCS SP5 confocal microscope with the Leica application suite 2.6.2 software. Images were processed and quantified using ImageJ software version 1.44p (National Institutes of Health).

### MMP-1 and TIMP quantitation

MMP-1 concentrations in cell culture supernatants were measured by Luminex (R&D Systems kit, Bio-Rad Luminex 200 machine). TIMP-1 and -2 concentrations were analyzed by ELISA (R&D Systems).

### Lung immunohistochemistry

Lung tissue from five patients with culture-proven *M. tuberculosis* was studied. Control tissue was obtained from uninvolved lung parenchyma of patients who underwent a surgical procedure for lung cancer. MT1-MMP immunoreactivity was evaluated using the mouse mAb 114-6G6 (Abcam, ab77965). Briefly, sections were rehydrated in graded alcohols and heated in a microwave oven at 900 W for 20 min in citrate buffer at pH 6. They were cooled at room temperature before immunostaining. MT1-MMP mAb was used at a concentration of 20 μg/ml for 2 h at room temperature and then processed with a polymer-HRP kit (BioGenex, San Ramon, CA) with diaminobenzidine development and Mayer hematoxylin counterstaining. Breast and colon tissues were used as a positive external control. Negative controls were obtained by omitting the primary Ab and using competing peptide used to raise the primary Ab. Slides were reviewed with a histopathologist, and images were taken using an Olympus BX51 microscope. This project was approved by the Hammersmith and Queen Charlotte’s Research Ethics Committee (reference no. 07/H0707/120).

### Chemical inhibitors and MT1-MMP inhibitory Ab

For chemical inhibition experiments, monocytes were preincubated with the specified inhibitors for 1 h prior to CoMTb stimulation. The p38 MAPK inhibitor SB203580 (Enzo Life Sciences) and ERK MAPK inhibitor PD98059 (Calbiochem) were used. The GPCR inhibitor pertussis toxin (Calbiochem) was used to block chemokine signaling. A mouse anti MT1-MMP Ab (MAB3328, Millipore) was used to inhibit MT1-MMP activity.

### Agarose bead assay

An agarose bead assay was used to investigate monocyte migration toward CoMTb using a modification of a previous assay (22). RPMI 1640 or CoMTb was added to a 0.5% agarose solution at 40°C at a 1:10 dilution. Beads (10 μl) were pipetted on to cover glass-bottom chamber slides (PAA Laboratories) precoated with type I collagen as described above. The slide was left at 4°C for 5 min for the agarose to set before adhering monocytes to the slide. Manganese (0.25 mM) was added to the media to activate integrins on the monocytes, which are required for monocyte adhesion to extracellular matrix during migration (23, 24). Images were captured using an Olympus E-620 camera attached to the Olympus CK2 light microscope. Images were processed and quantified using ImageJ software version 1.44p (National Institutes of Health). Migration was quantified by measuring four radial points per sphere and measuring the diameter of monocyte clusters, and two spheres per condition were analyzed.

### Statistical analysis

Statistical analysis was performed using GraphPad Prism 5 by a Student *t* test, Mann–Whitney *U* test, or one-way ANOVA with a Tukey post hoc analysis as appropriate. A *p* value < 0.05 was taken as significant.

## Results

### MT1-MMP expression is increased in sputum of patients with TB and correlates with lung infiltration

**FIGURE 1.**
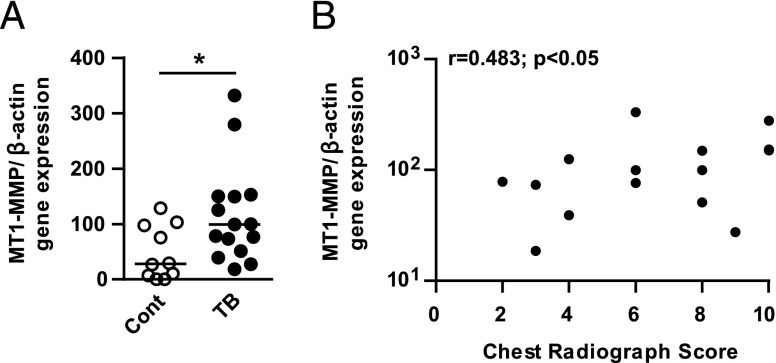
MT1-MMP gene expression is increased in the sputum of patients with TB and correlates with lung infiltration on chest radiographs. Induced sputum samples were collected prospectively from healthy controls (*n* = 10) and TB patients (*n* = 15) in Cape Town, South Africa. RNA was extracted from the sputum cell pellets, and MT1-MMP and β-actin gene expression were analyzed by RT-PCR. (**A**) MT1-MMP gene expression (normalized to β-actin) was increased in the sputum of TB patients compared with healthy controls. Horizontal line indicates median value. *p < 0.05 by Mann–Whitney *U* test. (**B**) The extent of lung infiltration on chest radiograph was scored on a scale of 0–10. Sputum MT1-MMP relative expression positively correlated with lung infiltration by Spearman correlation coefficient (*r* = 0.483, *p < 0.05).

We investigated MT1-MMP gene expression in induced sputum from patients with pulmonary TB (*n* = 15) and controls (*n* = 10). Clinical features of patient cohort are in Table I. The TB and control group were closely matched for age, but there was a higher proportion of females in the control group, which may reflect differences in health-seeking behavior (25). TB patients had significantly more symptoms (fever, cough, night sweats, weight loss, and breathlessness), a lower body mass index, and a higher number of abnormal respiratory examinations, consistent with the diagnosis of pulmonary TB. All control patients were smear and culture negative for *M. tuberculosis*. Twelve of the TB cases were culture positive and of the three remaining cases, one was smear positive. These three patients all had clinical and radiological features highly suggestive of TB and were started on TB therapy by their treating clinician. MT1-MMP gene expression normalized to β-actin was 5.1-fold higher in the sputum of patients with TB compared with controls (Fig. 1A, *p* < 0.05). MT1-MMP gene expression positively correlated with the extent of lung infiltration on chest radiograph (Fig. 1B; *r* = 0.483, *p* < 0.05).

**Table I.** Characteristics of patient cohort

Patient Characteristics	Control	TB	
Total no.	10	15	
Male/female	4/6	10/5	
Median age (range), years	31.5 (21–48)	40 (24–73)	NS
Symptoms (duration), days			
Any	1	15	
Fever	1	11	
Cough	0	14	
Hemoptysis	0	1	
Night sweats	1	12	
Weight loss	0	8	
Breathlessness	0	6	
Pleuritic pain	1	11	
Anorexia	1	3	
Median duration (range)	0 (0–4)	30 (7–98)	*p* < 0.0001
Examination			
Median body mass index (range)	25.4 (17.5– 55.7)	21.6 (18.5– 29.1)	*p* < 0.05
Temperature > 37.5°C	0	1	NS
Abnormal respiratory examination	4	13	
Smear			
Positive	0	8	
Negative	10	7	
Culture			
Positive	0	12	
Negative	10	3*^a^*	

Significant differences are indicated in the right-hand column. All patients had clinical features highly suggestive of TB with diagnostic features on chest radiograph and were commenced on antituberculous therapy by the treating physician.

^*a*^ One of these three patients was smear positive.

### *M. tuberculosis* infection of primary human monocytes drives MT1-MMP gene and protein expression

**FIGURE 2.**
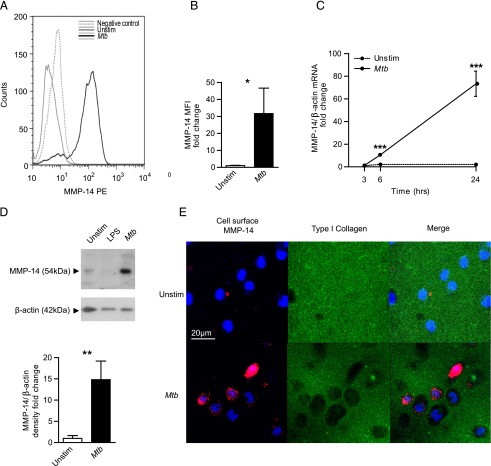
*M. tuberculosis* infection drives monocyte MT1-MMP expression and collagen degradation. (**A**) Human monocytes were infected with *M. tuberculosis* (MOI of 1) and MT1-MMP expression was analyzed by flow cytometry at 24 h. Increased MT1-MMP surface expression is demonstrated by greater mean fluorescence intensity (MFI). (**B**) MFI analysis confirms increased MT1-MMP expression. (**C**) *M. tuberculosis* infection causes a progressive increase in MT1-MMP mRNA accumulation infected monocytes (normalized to β-actin). (**D**) *M. tuberculosis* infection increases total cellular MT1-MMP at 48 h as analyzed by Western blotting, which was confirmed by densitometric analysis. β-actin was probed as a loading control. (**E**) *M. tuberculosis* infection causes collagen degradation around monocytes. Human monocytes were seeded on wells coated with fluorescently conjugated type I collagen and infected with *M. tuberculosis*. MT1-MMP surface expression and collagen degradation were analyzed by immunofluorescent staining and microscopy. Blue is DAPI nuclear stain, magenta is MT1-MMP, and green is type I collagen. Increased collagen degradation occurred at 24 h in *M. tuberculosis*–infected wells, and MT1-MMP surface expression colocalizes with the areas of collagen degradation. For (B)–(D), the mean ± SD values of experiments performed in triplicate are shown and are representative of a minimum of two independent experiments. *p < 0.05, **p < 0.01, ***p < 0.001 by Student *t* test.

In a primary human cellular model, *M. tuberculosis* infection increased monocyte MT1-MMP surface expression analyzed by flow cytometry compared with uninfected cells at 24 h (Fig. 2A). Quantification showed a 31.7-fold increase in MT1-MMP surface expression after *M. tuberculosis* infection (Fig. 2B, *p* < 0.05). Next, we analyzed the kinetics of MT1-MMP gene expression in *M. tuberculosis–*infected monocytes. *M. tuberculosis* infection caused a nonsignificant 1.4-fold increase in MT1-MMP gene expression normalized to β-actin at 3 h, rising to 10.6-fold at 6 h and 73.4-fold at 24 h relative to uninfected monocytes (Fig. 2C). Increased MT1-MMP mRNA accumulation was associated with increased MT1-MMP total protein in *M. tuberculosis*–infected monocytes analyzed by Western blotting (Fig. 2D). *M. tuberculosis* infection of monocytes caused a 14.8-fold increase in MT1-MMP protein in whole-cell lysates compared with uninfected monocytes at 48 h (Fig. 2D, *p* < 0.01). *M. tuberculosis* also increased secretion of MMP-1 into cell culture supernatants analyzed by Luminex array (Supplemental Fig. 1), although this concentration was below the sensitivity of detection by casein zymography (data not shown).

### *M. tuberculosis*–dependent MT1-MMP expression causes collagen degradation

**FIGURE 3.**
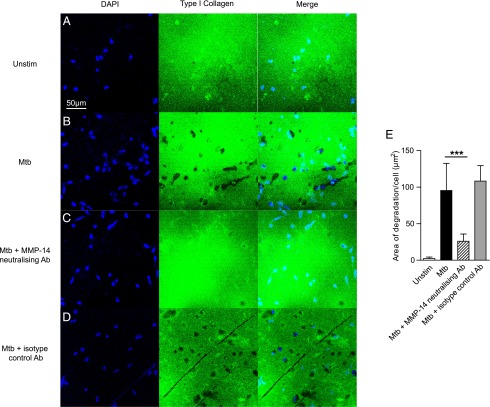
*M. tuberculosis*–driven collagen degradation is MT1-MMP–dependent. (**A** and **B**) Human monocytes were seeded on FITC-conjugated type I collagen and infected with *M. tuberculosis*. Collagen degradation was analyzed by immunofluorescent microscopy. Blue is DAPI nuclear stain; green is type I collagen. *M. tuberculosis* infection causes greater collagen degradation. (**C**) MT1-MMP activity was neutralized with an inhibitory anti-MT1-MMP Ab (MAB3328, Millipore) at 10 μg/ml. MT1-MMP inhibition inhibited areas of collagen breakdown. (**D**) An isotype control Ab did not inhibit collagen degradation. (**E**) Fluorescence quantitation confirms collagen degradation is MT1-MMP–dependent. Data are from a single experiment and are representative of three independent experiments. Mean values ± SD are shown. ***p < 0.001 by one-way ANOVA with a Tukey post hoc test.

To investigate whether MT1-MMP caused matrix breakdown, we analyzed degradation of fluorescent collagen. *M. tuberculosis* infection increased MT1-MMP surface expression on unpermeabalized monocytes at 24 h (Fig. 2E). *M. tuberculosis* infection caused collagen degradation, indicated by loss of fluorescence, and this collagen degradation colocalized with MT1-MMP expression (Fig. 2E, merged image). MT1-MMP neutralization inhibited such *M. tuberculosis*–driven collagen degradation. Zones of collagen breakdown are present around *M. tuberculosis*–infected monocytes (Fig. 3B), and incubation with an MT1-MMP inhibitory Ab suppresses this collagen breakdown (Fig. 3C). A matched isotype control Ab did not inhibit collage breakdown (Fig. 3D). Quantification of degraded collagen demonstrated that *M. tuberculosis* infection caused 42.8-fold greater collagen degradation by monocytes compared with uninfected cells (Fig. 3E). MT1-MMP inhibition resulted in a 73% reduction in this collagen breakdown (*p* < 0.001).

### MT1-MMP is expressed in granulomas of patients with TB and is upregulated by *M. tuberculosis*–induced intercellular networks

**FIGURE 4.**
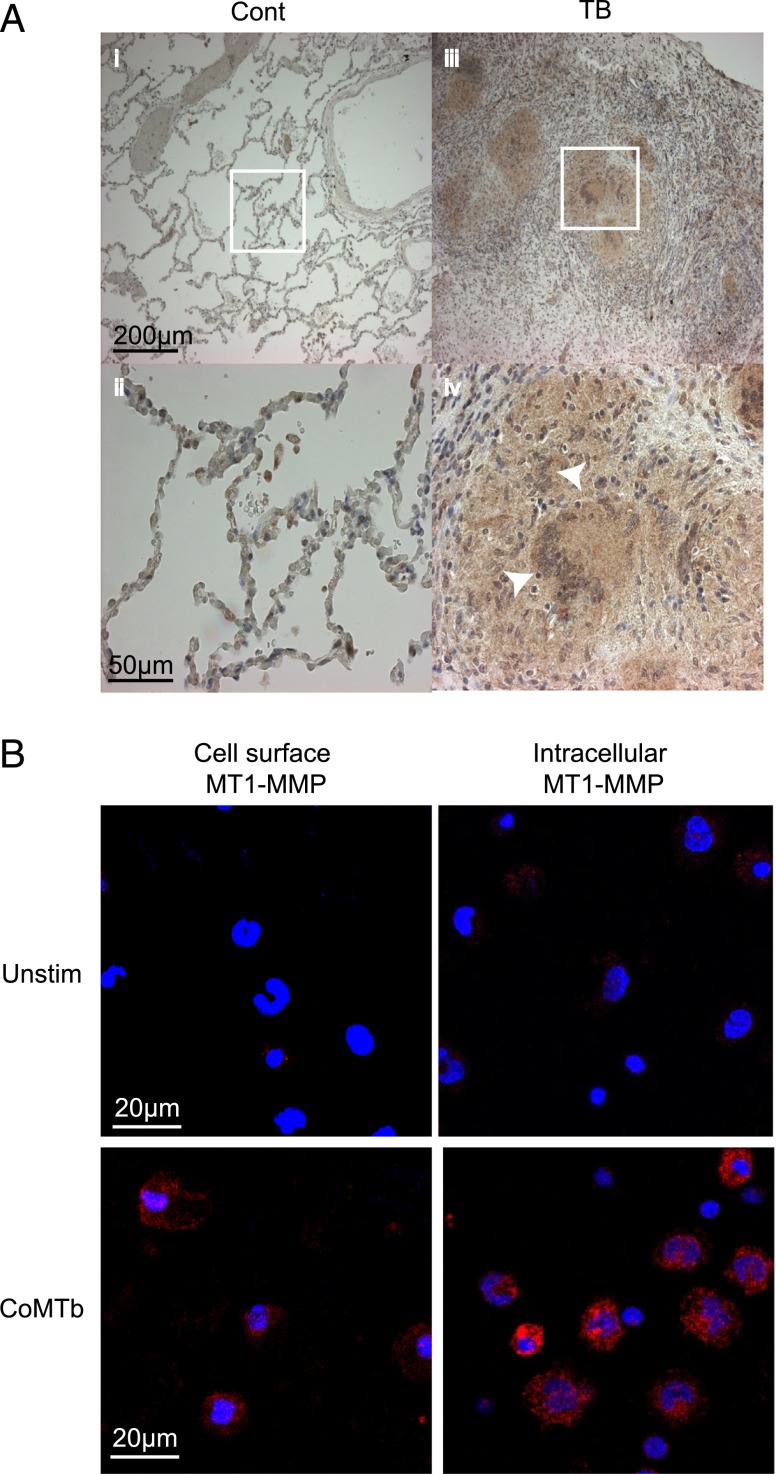
MT1-MMP is expressed in TB granulomas from patients and is driven by monocyte–monocyte networks. (**A**) Lung biopsies from five patients with culture-proven *M. tuberculosis* infection and control tissue from uninvolved lung parenchyma of patients with lung cancer were immunostained for MT1-MMP. In control biopsies, MT1-MMP immunostaining is present only in alveolar macrophages (i and ii). In TB, MT1-MMP immunoreactivity is present throughout the granuloma, expressed by both giant cells and epithelioid macrophages (iii and iv). White arrows indicate Langerhans multinucleate giant cells, surrounded by epithelioid macrophages. (**B**) Monocytes were stimulated with CoMTb to model intercellular networks. MT1-MMP expression was measured in unpermeabalized and permeabalized monocytes by immunofluorescent staining and microscopy. Blue is DAPI nuclear stain, magenta is MT1-MMP. CoMTb stimulation increased both surface and intracellular MT1-MMP expression at 24 h. Images are representative of three independent experiments.

Next, biopsies from patients with active TB and normal lung tissue were immunostained for MT1-MMP. MT1-MMP immunoreactivity was expressed in TB granulomas, including in Langerhans multinucleate giant cells and in surrounding epithelioid macrophages (Fig. 4A). In lung tissue of control subjects, MT1-MMP immunoreactivity was only detected in alveolar macrophages. Because MT1-MMP was diffusely expressed in the granuloma, but mycobacteria are relatively sparse within granulomas (26), we investigated whether *M. tuberculosis*–induced intercellular networks increased MT1-MMP expression. We stimulated cells with CoMTb to model these networks. CoMTb upregulated surface MT1-MMP expression in unpermeabalized and permeabilized monocytes at 24 h of incubation compared with unstimulated cells (Fig. 4B).

### *M. tuberculosis*–induced MT1-MMP expression is regulated by p38 MAPK and chemokine signaling

**FIGURE 5.**
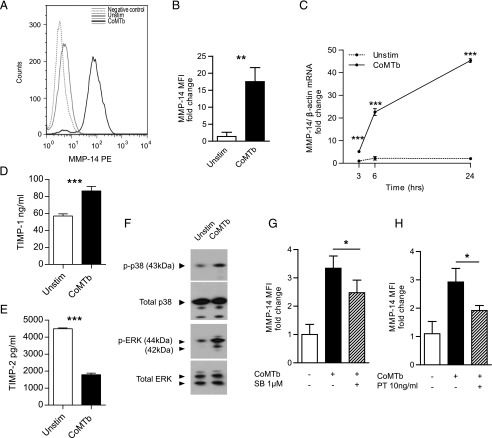
*M. tuberculosis*–driven monocyte networks upregulate MT1-MMP expression, regulated by p38 MAPK and GPCR signaling. (**A**) Human monocytes were stimulated with CoMTb, and MT1-MMP expression was analyzed at 24 h by flow cytometry. CoMTb upregulated MT1-MMP surface expression. (**B**) Analysis of fluorescence from three donors confirms increased monocyte surface MT1-MMP. (**C**) CoMTb causes a progressive increase in MT1-MMP mRNA relative to unstimulated monocytes. (**D**) Monocytes were stimulated with CoMTb and TIMP secretion was analyzed at 24 h. CoMTb increased TIMP-1 secretion. (**E**) Monocyte secretion of the specific MMP-14 inhibitor TIMP-2 is suppressed in CoMTb. (**F**) CoMTb stimulation increased p38 and ERK MAPK phosphorylation in monocytes at 30 min compared with unstimulated monocytes. Total p38 and ERK were unchanged. (**G** and **H**) Monocytes were preincubated with the p38 MAPK inhibitor SB203580 (SB) or pertussis toxin (PT) for 1 h prior to stimulation with CoMTb. MT1-MMP surface expression was measured by flow cytometry. Inhibition of p38 MAPK pathway (E) and GPCR signaling (F) downregulated CoMTb driven MT1-MMP expression at 24 h. The mean values ± SD are shown of experiments performed in triplicate on at least two occasions.*p < 0.05, **p < 0.01, ***p < 0.001 by Student *t* test.

Flow cytometry analysis demonstrated an increase in MT1-MMP mean fluorescence intensity in CoMTb-stimulated monocytes compared with unstimulated cells analyzed at 24 h (Fig. 5A), with a 17.5-fold increase in MT1-MMP surface expression with CoMTb (Fig. 5B, *p* < 0.05). CoMTb stimulation drove a 5.2-fold increase in MT1-MMP gene expression at 3 h, rising to 22.6-fold at 6 h and 45.4-fold at 24 h (Fig. 5C). CoMTb may contain and drive the production of inhibitors, so we analyzed TIMP-1 and -2 secretion from monocytes stimulated with CoMTb at 24 h. TIMP-1 concentrations were upregulated by CoMTb stimulation (Fig. 5D), but TIMP-2, the specific MT1-MMP inhibitor (27), was suppressed (Fig. 5E). MT1-MMP expression may be regulated by the MAPK pathways in response to other stimuli (28). CoMTb stimulation of monocytes caused a 3-fold increase in p38 phosphorylation and a 2.7-fold increase in ERK phosphorylation compared with unstimulated cells at 30 min incubation analyzed by Western blotting (Fig. 5F). Specific inhibition of the p38 MAPK signaling pathway by SB203580 resulted in reduced CoMTb-driven surface MT1-MMP expression on monocytes at 24 h (Fig. 5G, *p* < 0.05). In contrast, inhibition of the ERK MAPK pathway with PD98059 (10 μM) had no effect (data not shown).

Because CC chemokines are critical in monocyte migration to sites of infection, including TB (29), we measured chemokines in CoMTb from four different donors by Luminex array (Supplemental Table I). Multiple chemokine concentrations, including CCL2 (MCP-1), CCL3 (MIP-1α), CCL4 (MIP-1β), CXCL8 (IL-8), and CXCL9 (MIG), were increased. Therefore, we chemically inhibited all chemokine signaling via GPCRs with pertussis toxin to investigate whether chemokines in CoMTb were driving MT1-MMP expression. Pertussis caused a 35% reduction in CoMTb-driven MT1-MMP expression on unpermeabilized monocytes at 24 h (Fig. 5H, *p* < 0.05). Propidium iodide staining showed that pertussis did not increase cell death (data not shown). We stimulated monocytes with CCL-2 (MCP-1), which has previously been shown to upregulate MT1-MMP in endothelial cells (30), but CCL-2 did not increase monocyte MT1-MMP surface expression as a single stimulus.

### MT1-MMP is critical to monocyte migration

**FIGURE 6.**
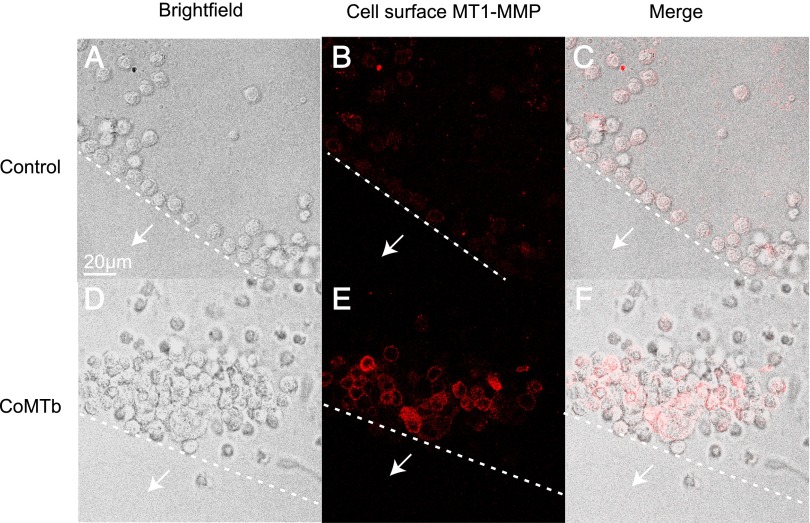
*M. tuberculosis*–induced intercellular networks stimulate monocyte migration. Agarose beads impregnated with RPMI 1640 (control) or CoMTb were set on slides coated with type I collagen. Monocytes were seeded on these slides. White arrows indicate the direction toward the bead center and dashed lines indicate the edge of the bead. MT1-MMP expression was measured in monocytes by immunofluorescent staining and microscopy. Magenta is MT1-MMP. (**A** and **D**) Light microscopy images show monocytes migrate toward the CoMTb bead at 24 h, forming clusters not observed for the control bead. (**B** and **E**) Surface MT1-MMP expression on monocytes migrating toward the CoMTb bead is greater than for the monocytes around the control bead. (**C** and **F**) Merged confocal and fluorescence images. Images shown are representative of three independent experiments.

**FIGURE 7.**
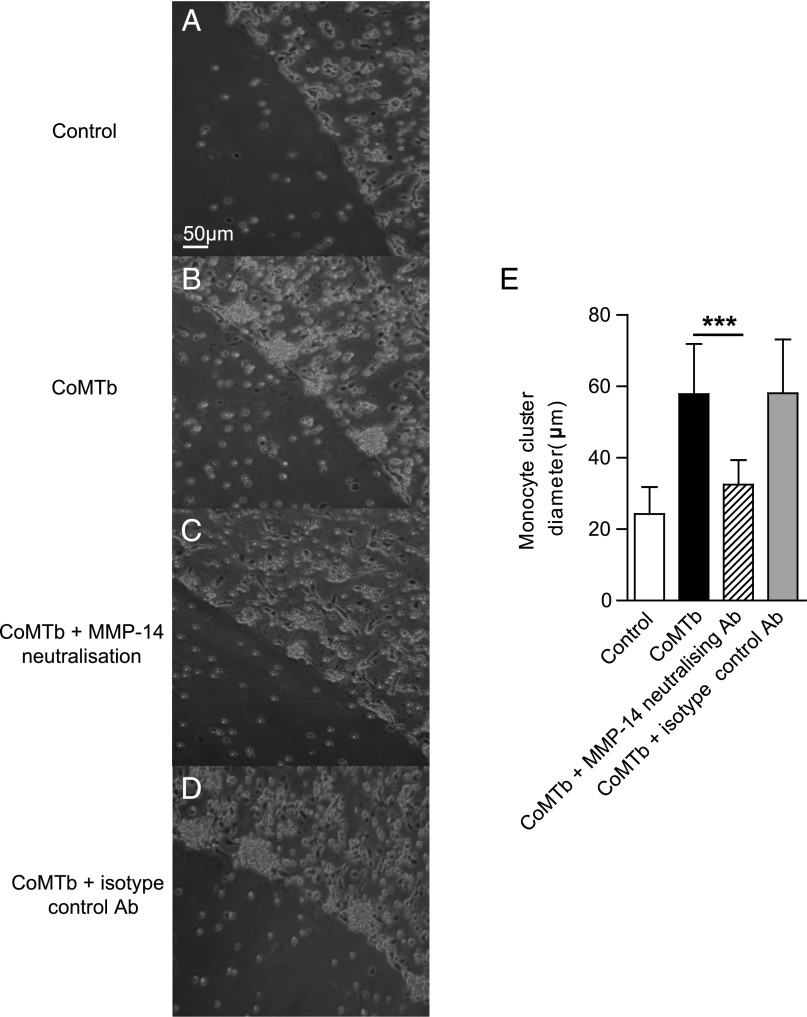
MT1-MMP is necessary for *M. tuberculosis*–driven monocyte migration. (**A** and **B**) Light microscopy images show migrating monocytes forming clusters around the CoMTb drop at 24 h. (**C**) Neutralization of MT1-MMP activity with an inhibitory anti–MT1-MMP Ab reduces monocyte migration. (**D**) An isotype control Ab did not affect cellular migration. (**E**) The maximum diameter of the monocyte clusters were analyzed, with images taken at four radial points per bead. Quantification confirms reduced monocyte migration with MT1-MMP neutralization. Data are means ± SD from a single experiment and are representative of two independent experiments. ***p < 0.001 by one-way ANOVA with a Tukey post hoc test.

Finally, we specifically investigated the role of MT1-MMP in monocyte migration using a modified agarose drop migration assay (22). Monocytes migrated toward CoMTb-impregnated agarose beads, forming clusters around the edge at 24 h that were not observed in control beads (Fig. 6A, 6D). The migrating monocytes expressed increased levels of membrane-associated MT1-MMP than cells around the control bead (Fig. 6B, 6E). To investigate the functional relevance, we specifically inhibited MT1-MMP activity with a neutralizing Ab. MT1-MMP inhibition reduced the size of monocyte clusters around beads compared with untreated cells (Fig. 7B, 7C). Isotype control Ab did not affect cell migration (Fig. 7D). Migration was quantified using two beads per condition, with monocyte migration measured at four radial points per sphere. The diameter of the monocyte clusters for CoMTb was twice that for the control bead (*p* < 0.005), and inhibition of MT1-MMP activity resulted in a 44% reduction in the diameter of the monocyte clusters (Fig. 7E, *p* < 0.005), whereas the matched isotype control Ab had no effect on monocyte migration toward CoMTb. Therefore, MT1-MMP is required for monocyte migration in this cellular model of TB.

## Discussion

We demonstrated in TB patients and in a human cell culture model that MT1-MMP expression in TB regulates both collagen destruction and cellular migration. MT1-MMP mRNA levels are increased in sputum of patients with active pulmonary TB, and MT1-MMP is expressed within granulomas of TB patients. *M. tuberculosis* infection and monocyte-dependent networks upregulate primary human monocyte expression of MT1-MMP and cause degradation of type I collagen, the main structural protein of the lung. Additionally, monocyte migration in TB is dependent on MT1-MMP, as shown by a novel agarose bead assay. MT1-MMP may play a pivotal role in the immunopathology of human TB by both degrading collagen and regulating cellular migration.

MT1-MMP has previously been implicated in TB pathogenesis by unbiased approaches. Gene expression profiling demonstrates that MT1-MMP is upregulated in *M. tuberculosis*–infected human macrophages (31), and a microarray study of human lung TB granulomas excised at surgery showed MT1-MMP gene expression to be 28.6-fold greater than in normal lung (19). Similarly, whole-blood gene expression profiling shows that MT1-MMP expression is higher in patients with active TB than in healthy controls (32) and MT1-MMP gene expression is elevated in nonhuman primate lungs 4 wk after TB infection (18). However, the functional significance of MT1-MMP activity has not previously been investigated. Multiple MMPs are emerging as important mediators in TB (11, 17, 20), and different MMPs may regulate specific aspects of immunopathology (33). The role of individual MMPs in TB pathology is likely to be both time- and cell-dependent. For example, MMP-9 derived from epithelial cells regulates cellular recruitment to the granuloma by inducing migratory gradients (11) whereas macrophage-derived MMP-1 is one effector of collagen destruction (17). MT1-MMP neutralization inhibited collagen destruction, suggesting that MT1-MMP is either the dominant collagenase in the immediate pericellular environment early in infection, consistent with other reports (12, 16, 34), or alternatively it is activating the proform of MMP-1 secreted by monocytes by enzymatic cleavage (27). MT1-MMP may regulate both cell migration and collagen destruction, and these processes may be co-dependent because collagen cleavage may be necessary for cells to migrate through the extracellular matrix.

We demonstrated that MT1-MMP colocalized with collagen destruction and that MT1-MMP neutralization could abrogate this proteolysis, implying a role in matrix destruction. Consistent with this, MT1-MMP causes matrix breakdown in other destructive pulmonary pathologies. MT1-MMP expression is upregulated in alveolar macrophages of smokers (35), and IL-13–driven emphysema in transgenic mouse models is associated with MT1-MMP upregulation (36). MT1-MMP is expressed in asthma and bronchiectasis, diseases that are characterized by pulmonary matrix remodeling (37). Other infectious conditions can upregulate MT1-MMP. For example, in hepatitis B–induced hepatocellular carcinoma, intrahepatic metastasis is driven by hepatitis B virus protein upregulating MT1-MMP (38). However, the role of MT1-MMP in bacterial infection has not been extensively studied.

We found that MT1-MMP was widely expressed in human TB granulomas, whereas bacilli are very sparse (26), leading us to investigate intercellular networks. Monocyte–monocyte signaling upregulated MT1-MMP expression and this was dependent on p38 MAPK and GPCR signaling. Although TIMP-1 was upregulated by *M. tuberculosis* infection, this does not inhibit MT1-MMP activity (27), whereas the specific inhibitor TIMP-2 was suppressed by monocyte intercellular networks, further skewing the local environment to matrix destruction. Consistent with our findings, p38 MAPK regulates MT1-MMP expression in fibronectin-stimulated macrophages (39). Studies in malignant cells have identified a role for ERK in driving constitutive MT1-MMP expression (40, 41), but we did not demonstrate ERK regulation of MT1-MMP in primary monocytes, indicating stimulus- and cell-specific regulation. We demonstrated that secretion of multiple chemokines is upregulated in *M. tuberculosis*–infected monocytes. Chemokines are critical to leukocyte migration to TB granulomas (42), but no single chemokine appears to be dominant due to redundancy within the signaling system. To perform global chemokine inhibition, we studied the effect of pertussis toxin, which inhibits GPCR signaling, and demonstrated that this suppressed CoMTb-driven MT1-MMP upregulation, suggesting a role for cytokines in upregulating MT1-MMP. Consistent with this, CCL2 stimulation of monocytes transmigrating through endothelium causes MT1-MMP clustering at motility-associated protrusions (43), and CCL2 and CXCL8 promote MT1-MMP surface expression, clustering, and activity in endothelial cells (30). However, in our system, CCL2 as a single stimulus did not upregulate total surface expression of MT1-MMP, suggesting that multiple factors may synergize to drive MT1-MMP. These may include either multiple host cytokines (44) or both host- and pathogen-secreted mediators as described for MMP-9 (11, 45).

Although the granuloma has traditionally been viewed as a host strategy to control mycobacterial growth, accumulating evidence suggests that monocyte migration may have deleterious effects to the host. In the *Mycobacterium marinum* zebrafish model, macrophages migrating into granulomas permit bacterial expansion and can disseminate infection (46). In mice, intranasal administration of poly(I:C), a type I IFN inducer, caused a CCR2-dependent excessive accumulation of monocytes in the lungs of *M. tuberculosis*–infected mice, leading to increased bacterial burden and decreased survival (47). However, whereas the chemotactic gradients driving the cellular recruitment have been dissected, the proteases driving leukocyte migration have not been identified (48). We demonstrate utilizing the agarose drop assay that MT1-MMP surface expression is driven by *M. tuberculosis*–induced intercellular networks and monocyte migration is MT1-MMP–dependent. Modulating MT1-MMP activity in TB may have diverse effects, for example by affecting intracellular signaling pathways regulated by MT1-MMP such as ERK and PI3K (49, 50). Therefore, investigating the global effect of MT1-MMP inhibition will require careful analysis in appropriate animal model systems.

MT1-MMP is critical to cellular migration in other pathologies (14, 16, 51), and in the agarose bead assay, inhibition suppressed cell migration, suggesting that it is the key collagenase in this process. MT1-MMP may be driving leukocyte migration by multiple mechanisms. First, MT1-MMP cleavages extracellular matrix at the leading edge of the cell, and also modification of cell surface proteins to promote the organized cell adhesion–de-adhesion required for migration (52, 53). Second, MT1-MMP–dependent cleavage of other cell surface proteins that interact with ECM components, such as CD44, integrins, and transglutaminase, are also considered important to migration of malignant cells (54–56). For example, MT1-MMP cleaves CD44 to produce a soluble fragment. Expression of MT1-MMP or CD44 in a breast cancer cell line alone did not stimulate cell migration, but coexpression did. Coexpression of a CD44 deletion mutant, which cannot be cleaved by MT1-MMP, resulted in loss of cell migration (56). Third, it has been postulated that MT1-MMP promotes cell migration by driving ERK MAPK phosphorylation. In a human fibrosarcoma cell line, TIMP-2 binding to MT1-MMP drove ERK signaling, which increased cell migration independently of the direct enzymatic activity of MT1-MMP (57).

In summary, we have demonstrated that MT1-MMP expression is increased in pulmonary TB and is functionally active, contributing to local tissue destruction and leukocyte recruitment to the granuloma. Therefore, MT1-MMP may represent a previously unrecognized regulator of these central immunopathological processes in human TB. Host-targeted therapies are emerging as a novel therapeutic paradigm in TB (58, 59), and the potential effect of such interventions on MMP-dependent cell migration and matrix destruction deserves evaluation.

## Supplementary Material

Data Supplement
