# Supplementary material for: Membrane Type 1 Matrix Metalloproteinase Regulates Monocyte Migration and Collagen Destruction in Tuberculosis
Source: J Immunol. 2015 Jun 19;195(3):882–91. doi: 10.4049/jimmunol.1403110 (PMC4505956; doi:10.4049/jimmunol.1403110)
Supplement: Data Supplement [file JI_1403110.zip › JI_1403110_Supplemental_Material_1.pdf]

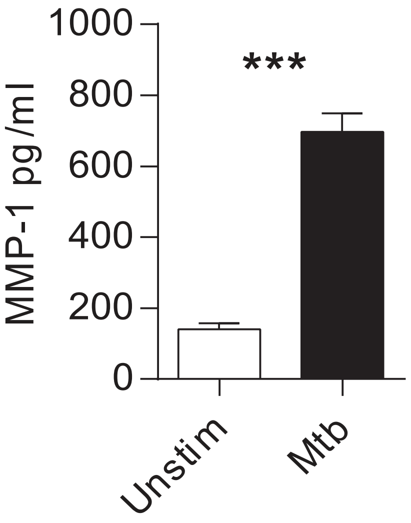

Supplementary figure 1: **MMP-1 secretion by Mtb-infected monocytes is increased at 24h.** Monocytes were infected with Mtb H37Rv at MOI 1 and supernatants harvested at 24h. MMP-1 concentration was analyzed by luminex. Mtb infection significantly increased MMP-1 secretion.

	CoMCont	CoMTb
**Cytokines**		
IL-1β	165 (39-192)	31813 (7994 - 39014)
IL-6	1 (1-1)	50914 (11903 - 63160)
TNFα	1 (1-1)	15369 (1607 - 63755)
IFNα	6 (6-6)	340 (69 - 550)
IL-12 (p40/p70)	2 (2-2)	752 (167 - 3853)
IL-1 receptor antagonist	628 (144-744)	8893 (3774 - 11242)
IL-2 receptor	2 (2-25)	994 (574 - 1326)
**Chemokines**		
CCL2 ((MCP-1)	6 (6-10)	1184 (334 - 2780)
CCL3 (MIP-1 α)	4 (4-4)	215660 (19320 - 361100)
CCL4 (MIP-1 β)	2 (2-2)	19799 (5630 -238242)
CXCL8 (IL-8)	34 (9-41)	96439 (7176 - 180980)
CXCL9 (MIG)	47 (8-86)	175 (50 - 217)
**Growth factors**		
GM-CSF	3 (3-3)	286 (70-1324)
VEGF	35 (7-45)	168 (95 - 192)
G-CSF	3 (3-3)	1394 (245 - 1896)
FGF	256 (64 - 259)	343 (94 - 378)

Supplementary table 1: Cytokines, chemokines and growth factors in CoMCont and CoMTb.

Median levels and interquartile ranges (in brackets) as shown. Units pg/ml. Abbreviations: CoMCont – Conditioned medium from control uninfected monocytes. CoMTb – Conditioned Medium from *Mtb* infected monocytes. Additional analytes that were measured by luminex array that did not significantly change between conditions were IFN-γ, IL-2, IL-4, IL-5, IL-7, IL-10, IL- 13, IL-15, IL-17, CCL-5 (RANTES), CXCL-10 (IP-10), CCL-11 (Eotaxin), EGF and HGF.
